# Supplementary material for: Cross-Reactivity Conferred by Homologous and Heterologous Prime-Boost A/H5 Influenza Vaccination Strategies in Humans: A Literature Review
Source: Vaccines (Basel). 2021 Dec 10;9(12):1465. doi: 10.3390/vaccines9121465 (PMC8708856; doi:10.3390/vaccines9121465)
Supplement: Supplementary file 1 [file vaccines-09-01465-s001.zip › Figure S1.html]

Supplementary figure 1


Supplementary figure 1

Kok, A.; Fouchier, R.; Richard M. Cross-reactivity conferred by homologous and heterologous prime-boost A/H5 influenza vaccination strategies in humans: a literature review. *Vaccines* **2021**.

**Supplementary figure 1: Interactive display of immunological endpoints upon homologous vaccination regimens** Immunological endpoint values obtained against the homologous antigen (x-axis) are plotted against those obtained against heterologous antigens (y-axis). Information on each specific datapoint can be visualized by hovering over the datapoint. In tab **I**, the color and fill of the individual points indicate the vaccine type and adjuvant combination according to the legend and the size of the circles indicates the number of vaccine doses (one to four) used before the serum sample was obtained according to the legend. In tab **II**, the fill of the individual points indicate the amount of HA present in the vaccine according to the legend. In tab **III**, the fill of the individual points indicate the vaccine antigen used according to the legend.

# I. Vaccine, Adjuvant, Nb. of doses

## Column

### % Seroprotection (HI)

### % Seroprotection (MN)

## Column

### % Seroconversion (HI)

### % Seroconversion (MN)

## Column

### Log 2 GMT (HI)

### Log 2 GMT (MN)

# II. Amount of HA

## Column

### % Seroprotection (HI)

### % Seroprotection (MN)

## Column

### % Seroconversion (HI)

### % Seroconversion (MN)

## Column

### Log 2 GMT (HI)

### Log 2 GMT (MN)

# III. Vaccine antigen

## Column

### % Seroprotection (HI)

### % Seroprotection (MN)

## Column

### % Seroconversion (HI)

### % Seroconversion (MN)

## Column

### Log 2 GMT (HI)

### Log 2 GMT (MN)
