# Supplementary material for: Cross-Reactivity Conferred by Homologous and Heterologous Prime-Boost A/H5 Influenza Vaccination Strategies in Humans: A Literature Review
Source: Vaccines (Basel). 2021 Dec 10;9(12):1465. doi: 10.3390/vaccines9121465 (PMC8708856; doi:10.3390/vaccines9121465)
Supplement: Supplementary file 1 [file vaccines-09-01465-s001.zip › Figure S2.html]

Supplementary figure 2


Supplementary figure 2

Kok, A.; Fouchier, R.; Richard M. Cross-reactivity conferred by homologous and heterologous prime-boost A/H5 influenza vaccination strategies in humans: a literature review. *Vaccines* **2021**.

**Supplementary figure 2: Interactive display of immunological endpoints upon heterologous vaccination regimens** Immunological endpoint values obtained against the antigen homologous to the primary vaccine strain (x-axis) are plotted against those obtained against the antigen homologous to the secondary vaccine strain (y-axis). Information on each specific datapoint can be visualized by hovering over the datapoint. In tab **I**, the color and fill of the individual points indicate the vaccine type and adjuvant combination in the primary vaccination according to the legend and the size of the circles indicates the number of vaccine doses used before the serum sample was obtained according to the legend. In tab **II**, the fill of the individual points indicate the amount of HA present in the primary vaccination according to the legend. In tab **III**, the fill of the individual points indicate the amount of HA present in the secondary vaccination according to the legend. In tab **IV**, the fill of the individual points indicate the vaccine antigen used in the primary vaccination according to the legend. In tab **V**, the fill of the individual points indicate the vaccine antigen used in the secondary vaccination according to the legend.

# I. Vaccine, Adjuvant, Nb. of doses

## Column

### % Seroprotection (HI)

### % Seroprotection (MN)

## Column

### % Seroconversion (HI)

### % Seroconversion (MN)

## Column

### Log 2 GMT (HI)

### Log 2 GMT (MN)

# II. Amount of HA primary vaccination

## Column

### % Seroprotection (HI)

### % Seroprotection (MN)

## Column

### % Seroconversion (HI)

### % Seroconversion (MN)

## Column

### Log 2 GMT (HI)

### Log 2 GMT (MN)

# III. Amount of HA secondary vaccination

## Column

### % Seroprotection (HI)

### % Seroprotection (MN)

## Column

### % Seroconversion (HI)

### % Seroconversion (MN)

## Column

### Log 2 GMT (HI)

### Log 2 GMT (MN)

# IV. Vaccine antigen primary vaccination

## Column

### % Seroprotection (HI)

### % Seroprotection (MN)

## Column

### % Seroconversion (HI)

### % Seroconversion (MN)

## Column

### Log 2 GMT (HI)

### Log 2 GMT (MN)

# V. Vaccine antigen secondary vaccination

## Column

### % Seroprotection (HI)

### % Seroprotection (MN)

## Column

### % Seroconversion (HI)

### % Seroconversion (MN)

## Column

### Log 2 GMT (HI)

### Log 2 GMT (MN)
