# Supplementary material for: Cross-Reactivity Conferred by Homologous and Heterologous Prime-Boost A/H5 Influenza Vaccination Strategies in Humans: A Literature Review
Source: Vaccines (Basel). 2021 Dec 10;9(12):1465. doi: 10.3390/vaccines9121465 (PMC8708856; doi:10.3390/vaccines9121465)
Supplement: Supplementary file 1 [file vaccines-09-01465-s001.zip › Figure S3.html]

Supplementary figure 3


Supplementary figure 3

Kok, A.; Fouchier, R.; Richard M. Cross-reactivity conferred by homologous and heterologous prime-boost A/H5 influenza vaccination strategies in humans: a literature review. *Vaccines* **2021**.

**Supplementary figure 3: Longevity of immunological endpoints upon homologous and heterologous vaccination regimens.** Immunological endpoint values (y-axis) are plotted against the number of days since the last vaccine administration (x-axis). Information on each specific datapoint can be visualized by hovering over the datapoint. Data from homologous vaccination regimens are depicted in tab **I** (HI) and **II** (MN). The immunological endpoints against the vaccine antigens are plotted in the top row and those against the heterologous antigens in the bottom row. Data from heterologous vaccination regimens are depicted in tab **III** (HI) and **IV** (MN). The immunological endpoints against the primary vaccine antigens are plotted in the top row and those against the secondary vaccine antigens in the bottom row.

# I. Homologous vaccination (HI)

## Column

### % Seroprotection (HI) vaccine antigen

### % Seroprotection (HI) heterologous antigen

## Column

### % Seroconversion (HI) vaccine antigen

### % Seroconversion (HI) heterologous antigen

## Column

### Log 2 GMT (HI) vaccine antigen

### Log 2 GMT (HI) heterologous antigen

# II. Homologous vaccination (MN)

## Column

### % Seroprotection (MN) vaccine antigen

### % Seroprotection (MN) heterologous antigen

## Column

### % Seroconversion (MN) vaccine antigen

### % Seroconversion (MN) heterologous antigen

## Column

### Log 2 GMT (MN) vaccine antigen

### Log 2 GMT (MN) heterologous antigen

# III. Heterologous vaccination (HI)

## Column

### % Seroprotection (HI) primary vaccine antigen

### % Seroprotection (HI) secondary vaccine antigen

## Column

### % Seroconversion (HI) primary vaccine antigen

### % Seroconversion (HI) secondary vaccine antigen

## Column

### Log 2 GMT (HI) primary vaccine antigen

### Log 2 GMT (HI) secondary vaccine antigen

# IV. Heterologous vaccination (MN)

## Column

### % Seroprotection (MN) primary vaccine antigen

### % Seroprotection (MN) secondary vaccine antigen

## Column

### % Seroconversion (MN) primary vaccine antigen

### % Seroconversion (MN) secondary vaccine antigen

## Column

### Log 2 GMT (MN) primary vaccine antigen

### Log 2 GMT (MN) secondary vaccine antigen
